# Comparing the effectiveness of two surgical techniques for treating lower lid epiblepharon in children: a randomized controlled trial

**DOI:** 10.1038/s41598-023-32050-4

**Published:** 2023-04-11

**Authors:** Masaki Takeuchi, Nozomi Matsumura, Tomoko Ohno, Takeshi Fujita, Mizuki Asano, Nobuhisa Mizuki

**Affiliations:** 1 Department of Ophthalmology and Visual Science, Yokohama City University, 3-9 Fukuura, Kanazawa-ku, Yokohama, Kanagawa 236-0004 Japan; 2 Department of Ophthalmology, Kanagawa Children’s Medical Center, 2-138-4 Mutsukawa, Minami-ku, Yokohama, Kanagawa 232-0066 Japan

**Keywords:** Eyelid diseases, Eye abnormalities

## Abstract

A multicenter randomized controlled trial was conducted to compare the effectiveness of incisional and nonincisional surgical techniques for treating lower lid epiblepharon in children. The study included 89 eyes from 50 children aged 3–15 years (mean, 7.5 ± 2.4 years) with moderate lower lid epiblepharon. Patients were randomly assigned to either incisional (modified Hotz procedure with lid margin splitting; 45 eyes of 25 patients) or nonincisional (44 eyes of 25 patients) surgery groups. Treatment outcomes and changes in astigmatism were evaluated 6 months after surgery. Incisional surgery provided a significantly higher percentage (77.8%) of well-corrected treatment results (*P* = 0.026; odds ratio, 2.88; 95% confidence interval, 1.07–8.22) than nonincisional surgery (55.4%). The mean change in astigmatism 6 months after surgery was − 0.24 ± 0.42 and − 0.01 ± 0.47 D in the incisional and nonincisional surgery groups, respectively. The improvement in astigmatism was significantly higher in the incisional surgery group than in the nonincisional surgery group (*P* = 0.008). The incisional surgical treatment for moderate epiblepharon in children resulted in a higher number of well-corrected patients, indicating an absence of both ciliary touch and superficial keratitis as well as statistically significant improvements in astigmatism correction.

## Introduction

Epiblepharon is defined as a fold of skin that stretches across the edge of the eyelid and presses the lashes against the globe^1^. It usually affects the lower lid and is a common condition among East Asian children. This can lead to the manifestation of several symptoms, such as tearing, discharge, photophobia, irritation, foreign body sensation, and visual disturbances caused by corneal injury. The initial treatment is conservative; however, it may occasionally require surgical correction^1–4^.

A variety of surgical techniques have been suggested for treating patients with epiblepharon. Among these, nonincisional suturing and incisional surgical techniques (modified Hotz procedure) have been reported as the primary representative surgeries^1,4,5^. However, to the best of our knowledge, no randomized controlled trials have been conducted to evaluate and compare the effectiveness of nonincisional suturing versus incisional surgical techniques.

Astigmatism is more common in patients with epiblepharon compared with those without epiblepharon^2,6^. Sohn et al.^5^ reported that the average value of astigmatism among Korean children was 1.52 D in those with epiblepharon and 0.73 D in those without epiblepharon. The effect of surgical correction on reducing the extent of astigmatism in patients with epiblepharon is controversial^2,6–11^. Although changes in astigmatism are observed after the surgical correction of epiblepharon, it is unclear whether any difference in astigmatism reduction exists between different techniques. Thus, this study aimed to compare the effectiveness and astigmatic changes of two representative surgical techniques for treating pediatric epiblepharon.

## Subjects and methods

The present study was conducted according to the tenets of the Declaration of Helsinki. The research protocol and informed consent/assent forms were approved by the institutional review board of Yokohama City University Hospital, and Kanagawa Children’s Medical Center. Written informed consent was obtained from each parent or guardian of the patients included in the study as well as patients aged more than 6 years who were capable of understanding the purpose of this study. Informed consent for publication of identifying information/images in an online open-access publication was also obtained from all patients and/or their legal guardian. This study was supervised by an independent data and safety monitoring committee. This study is available on the website of UMIN Clinical Trial Registry (www.umin.ac.jp/UMIN000027321, accessed 5/12/2017).

### Patient selection

**Figure 1:**
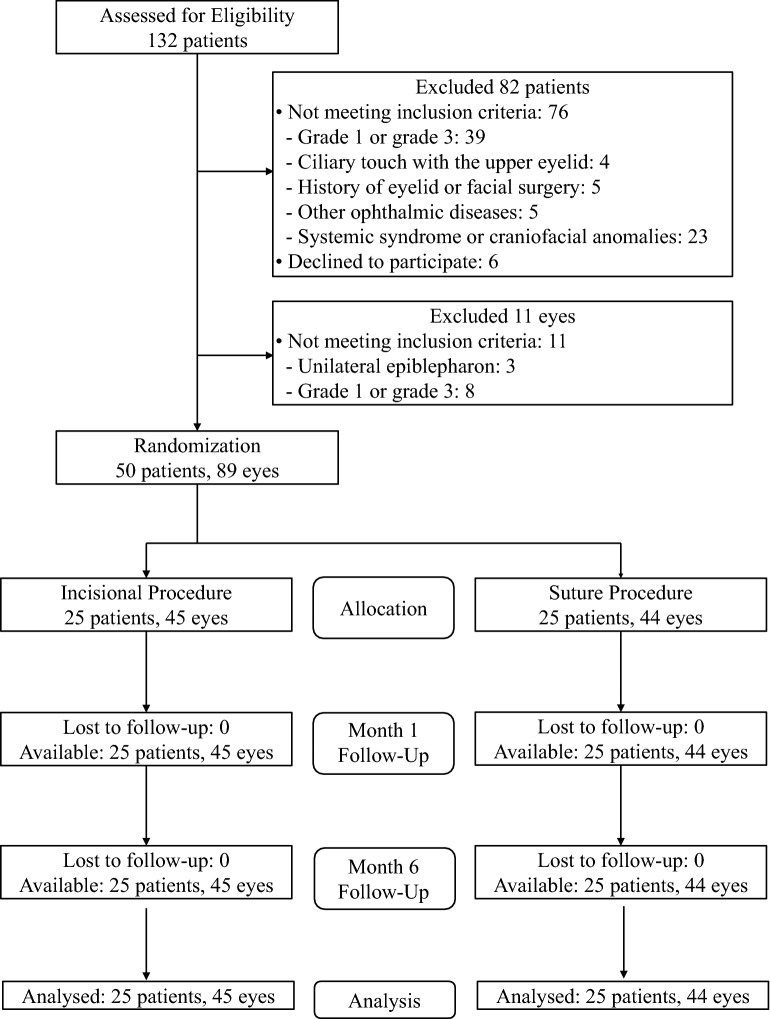
CONSORT diagram.

Patients were selected for the study between September 2017 and July 2019 at Yokohama City University Hospital, Yokohama, Japan, and Kanagawa Children’s Medical Center, Yokohama, Japan (Fig. 1). Patients diagnosed with lower lid epiblepharon with keratitis aged 3–15 years and approval of parents or guardians regarding surgical treatment options were the main eligibility criteria for inclusion in this study. Ciliary touch on the cornea was graded from 1 to 3, as shown in the Supplementary Fig. S1. In this study, only moderate grade 2 cases were included.

Patients with a history of eyelid or facial surgery, ciliary touch on the upper eyelid, ptosis, and/or abnormalities in eyelid opening or closing as well as those with systemic syndrome or craniofacial anomalies and other ophthalmic diseases such as corneal opacity were excluded from the study.

### Randomization

The patient registration numbers were sent to a data center, which randomly assigned each patient (using random numbers generated by Excel) to one of the two surgical treatment groups with equal probability: (1) incisional surgery group (modified Hotz procedure with lid margin splitting [LMS]) or (2) nonincisional surgery group (buried sutures).

### Examinations and follow-up

Randomization and preoperative examination were performed 1 month before the surgery. The examination included visual acuity, refractive examination using an auto refractometer, slit-lamp examination with fluorescent staining, and capturing the eyelid photographs. After surgery, both groups were followed up at 4 ± 1 weeks and 26 ± 4 weeks. At both follow-up visits, the same examinations were performed as those before the surgery. The primary and secondary outcomes were evaluated 6 months after the surgery.

### Surgical techniques

General anesthesia was used for all surgical procedures. One of the two ophthalmologists performed the surgery, and both surgeons performed identical surgeries, as described in the methods below. The incision method (modified Hotz procedure with LMS) was based on the previously described method by Hwang et al.^12^ LMS was performed first in all cases of incisional surgical techniques. A 1-mm deep incision was made along the gray line using a no. 15 scalpel blade after grasping the lower lid with chalazion forceps. Following the horizontal extent of the epiblepharon, the split extended from 1 mm lateral to the punctum and the medial third or half of the lower eyelid. Between the orbicularis skin flap and the tarsus, the subsidiary skin was incised and dissected.

The subcutaneous tissue of the orbicularis skin flap was fixed to the lower margin of the tarsus with three interrupted sutures using 6–0 polyglactin (Vicryl; Ethicon Inc, Somerville, NJ). Skin edges were closed using 8–0 polyglactin (Vicryl), and sutures were not removed.

The nonincisional technique (buried sutures) was based on the method described by Seo et al.^13^. A double-armed 7–0 polyester suture was used. Before suturing, a small incision was made at the exit site of the suture needle. First, sutures were passed from the inferior cul-de-sac and out approximately 3–4 mm below the eyelid margin. The conjunctival side was threaded subconjunctivally, whereas the dermal side was threaded subcutaneously, both pointing a few millimeters laterally. The thread from the conjunctival side was brought out to the same location as the thread from the dermal side, where it was tightly tied and buried. Two sutures were inserted into the lower eyelids; one in the medial and the other in the central region. The buried sutures were not removed.

### Clinical outcomes

The primary endpoint was treatment success, i.e., percentage of well-correction, which is defined as the absence of both ciliary touch and superficial keratitis assessed 6 months after surgery. The treatment outcomes were graded on three levels: well-corrected (no ciliary touch or superficial keratitis); under-corrected (ciliary touch and superficial keratitis); and over-corrected (eyelid ectropion and insufficient eyelid closure).

The secondary endpoint included the assessment and comparison of pre- and postoperative changes in astigmatism for both techniques using an auto refractometer. As a safety assessment, the presence of adverse events was also examined.

### Statistical methods

Based on our previous surgical outcomes, we calculated the sample size. A retrospective analysis of our previous surgical outcomes (with a 3-month follow-up period) revealed that the clinical success rate of the suture method was 63% and that of the incision method was 90%. The sample size was then calculated using the following conditions based on these findings: Fisher’s exact test, α error of 0.05, and statistical power of 0.8. The required number of eyes in each group was 45. The success rate was evaluated using Fisher’s exact test. Wilcoxon’s signed-rank test was used to assess the changes in astigmatism. The Mann–Whitney U test was used to compare the groups in terms of astigmatism changes before and after surgery. A *P*-value of ≤ 0.05 was considered statistically significant. The EZR software was used for all analyses (Saitama Medical Center, Jichi Medical University).

## Results

This study included 89 eyes from 50 patients (22 males and 28 females) of the 132 patients who were evaluated for eligibility. The mean age of the patients was 7.5 years (range: 3–12 years). The incision method was assigned to 25 patients with 45 eyes at random, whereas the suture method was assigned to 25 patients with 44 eyes. The incision method was assigned to 8 (32%) males and 17 (68%) females, while the suture method was assigned to 14 (56%) males and 11 (44%) females.

No statistically significant difference was observed in terms of age, sex, and preoperative astigmatism between the two groups (Table 1). All 50 patients completed 26 ± 4 weeks of follow-up, and no patient dropped out during the study.

**Table 1:** Characteristics of patients enrolled in this study.

Variables	Incisional technique	Suture technique	
Number of cases	25	25	
Sex (M/F)	8/17	14/11	n.s
Year-of-age, months ± SD	7.9 (2.4)	7.0 (2.3)	n.s
Astigmatism, diopter ± SD	1.21 (1.06)	1.48 (1.20)	n.s

n.s., not significant.

At 1 month after surgery, the incisional surgery group revealed well-corrected, under-corrected, and over-corrected treatment results in 43 (95.6%), 2 (4.4%), and 0 (0%) eyes, respectively (Table 2). Similarly, the nonincisional surgery group revealed well-corrected, under-corrected, and over-corrected treatment results in 38 (86.4%), 6 (13.6%), and 0 (0%) eyes, respectively (Table 2). In terms of the percentage of well-corrected outcomes, no statistically significant difference was observed in surgical outcomes 1 month after surgery between the two groups.

**Table 2:** Surgical outcome at 1-month and 6-month follow-up after epiblepharon surgery.

Methods	Well-corrected	Under-corrected	Over-corrected	*P*-value	Odds ratio (95% CI)
1-month follow-up					
Incisional (%)	43 (95.6)	2 (4.4)	0 (0)	0.16	3.35 (0.56–35.9)
Suture (%)	38 (86.4)	6 (13.6)	0 (0)		
6-month follow-up					
Incisional (%)	35 (77.8)	10 (22.2)	0 (0)	0.026	2.88 (1.07–8.22)
Suture (%)	24 (54.5)	20 (45.5)	0 (0)		

At 6 months after surgery (primary endpoint), the incisional surgery group revealed well-corrected, under-corrected, and over-corrected treatment results in 35 (77.8%), 10 (22.2%), and 0 (0%) eyes, respectively (Table 2). Likewise, the nonincisional surgery group revealed well-corrected, under-corrected, and over-corrected treatment results in 24 (54.5%), 20 (45.5%), and 0 (0%) eyes, respectively (Table 2). The incisional surgery group had statistically significantly better outcomes than the nonincisional surgery group (*P* = 0.026; odds ratio [OR], 2.88; 95% confidence interval [CI], 1.07–8.22).

In the incisional group, the mean preoperative astigmatism was 1.21 ± 1.06 D, and the mean postoperative astigmatism was 0.97 ± 1.04 D (Table 3).

**Table 3:** Changes in astigmatism at 6-month follow-up after epiblepharon surgery.

Methods	Preoperative, diopter ± SD	Postoperative, diopter ± SD	Difference, diopter ± SD	*P*-value
Incisional (n = 45)	1.21 (1.06)	0.97 (1.04)	− 0.24 (0.42)	< .001
Suture (n = 44)	1.48 (1.20)	1.48 (1.40)	− 0.01 (0.47)	0.86

A statistically significant decrease was observed in astigmatism after surgery (*P* < 0.001). In the nonincisional surgery group, the mean preoperative astigmatism was 1.48 ± 1.20 D, and the mean postoperative astigmatism was 1.48 ± 1.40 D (*P* = 0.857). The mean change in astigmatism was − 0.24 ± 0.42 D in the incisional surgery group and − 0.01 ± 0.47 D in the nonincisional surgery group. The improvement in astigmatism at 6 months after surgery was statistically significant in the incisional surgery group compared with the nonincisional surgery group (*P* = 0.008) (Supplementary Fig. S2).

The following two adverse events were recorded as part of a safety assessment. A 6-year-old boy who had incisional surgery in both eyes developed a chalazion in the right eye 11 weeks later, which coincided with the incision site at the lower eyelid. Conservation treatment with antibiotic drops and ointment resulted in the resolution of chalazion within 2 weeks without scarring. Additionally, 12 weeks after the bilateral lower eyelid suture surgery, a 4-year-old boy was found to have herpes simplex in the upper and lower eyelids of both eyes. Conservative dermatological treatment with antiviral ointment resulted in the resolution of herpes lesions within 2 weeks without scarring. In both cases, the relationship between the surgery and symptoms is unclear. No other adverse events were reported. Supplementary Fig. S3 shows the representative photographs of both surgeries before surgery and 6 months after. No significant complications or complaints from patients or their parents were noted regarding scarring on the eyelids after either of surgical techniques.

## Discussion

We conducted the first randomized clinical trial (RCT) of epiblepharon to accurately evaluate the effectiveness and safety of incisional and suture techniques. In this study, only patients with grade 2 moderate epiblepharon, following the classification proposed by Lee et al.^11^, were included. In our facilities, grade 1 mild epiblepharon often resolves spontaneously without surgery, whereas some patients with grade 3 epiblepharon need to be treated with an incisional technique via epicanthoplasty. Therefore, patients with grade 2 epiblepharon are considered suitable candidates for either incisional or suture techniques.

At 6 months after surgery, surgical outcomes in this study were classified as well-corrected in 77.8% of cases of incision and 54.5% of cases of suture, confirming the effectiveness of the techniques for treating epiblepharon. According to previous studies, the recurrence rate for incision techniques was 0%–9.1%^1,4,12,14^ and that for suture techniques was 7.3–44.4%^1,13,15,16^. The proportions of well-corrected outcomes in the two groups were slightly lower than the treatment success rates in previous studies. Previous reports, however, provided a varying definition of cure and recurrence. Only patients with no ciliary touch and no keratitis were considered to have well-corrected outcomes in this study. These stringent criteria could explain why there are so few well-corrected cases in both surgical procedures in the present study compared with those in previous studies.

This study revealed that the incisional technique was statistically superior to the suture technique in terms of the number of cases with well-corrected outcomes at 6-month follow-up. Although there have been several reports on these two major surgical techniques, the differences in clinical outcomes between the two techniques and the indication of the surgical techniques have rarely been evaluated. Sunder et al. retrospectively reviewed the two techniques; however, no statistical analysis was performed due to the small number of cases^1^. Therefore, the findings of this prospective study with randomized allocation, and the application of clear classification and evaluation criteria regarding the effects of the two surgical techniques are essential for evidence-based decisions for treating lower eyelid epiblepharon.

Refractory corneal epithelial lesions caused by epiblepharon can induce corneal astigmatism and result in amblyopia in severe cases. The effect of surgical correction on reducing the extent of astigmatism in patients with epiblepharon is controversial^2,6–10,17^. This randomized controlled trial demonstrated a significant improvement in astigmatism via incision surgery, whereas no change was observed via the suture techniques. However, it is debatable whether the incision method’s minor improvement in astigmatism benefits the patient clinically. Further studies on the effect of reduced lid epiblepharon surgery on astigmatism advancements are expected.

Compared with the incision technique, the suture technique is minimally invasive and requires less operative time; therefore, performing surgery under local anesthesia on young patients might be possible. Thus, the suture technique may be appropriate for treating mild cases and surgery under local anesthesia.

This study has several limitations. First, this RCT included only patients with moderate epiblepharon (Grade II). Hence, the results of this research portray moderate epiblepharon rather than all epiblepharon. LMS, which is considered effective in reducing recurrence, was performed in all cases for the incision technique^14^. It has been suggested, in particular, that in cases involving epicanthus, the vertical running of skin and orbicularis oculi as well as epicanthus can cause strong traction, inhibiting the tissue adhesions formed by surgery and contributing to the recurrence of epiblepharon^18^. However, epicanthoplasty, another optional procedure for the incision technique, was not performed^19–21^. As these procedures could influence the measured outcomes, further studies are required to evaluate the indications for LMS and epicanthoplasty. Although there was worry about the impact of active accommodation on refractive error, including astigmatism, in children, cycloplegic autorefraction was not conducted in all patients in this research. As for the follow-up period, our observational study was conducted with a 6-month follow-up based on the hypothesis that most recurrences occur within 6 months^15^. However, a longer observational period might be necessary to identify and evaluate potential recurrences.

## Conclusion

At 6 months of follow-up, this is the first RCT comparing two surgical techniques for treating moderate lower eyelid epiblepharon in children, and it found that the incision technique resulted in a greater number of well-corrected patients compared with the suture technique.

## Supplementary Information

Supplementary Information.

## Data Availability

The trial protocol and the data supporting the findings of this study are available from the corresponding author upon reasonable request.
