# Supplementary material for: Comparing the effectiveness of two surgical techniques for treating lower lid epiblepharon in children: a randomized controlled trial
Source: Sci Rep. 2023 Apr 11;13:5857. doi: 10.1038/s41598-023-32050-4 (PMC10090162; doi:10.1038/s41598-023-32050-4)
Supplement: Supplementary file 1 — Supplementary Information. [file 41598_2023_32050_MOESM1_ESM.docx]

**Supplementary Information**

Comparing the Effectiveness of Two Surgical Techniques for Treating Lower Lid Epiblepharon in Children: A Randomized Controlled Trial

Masaki Takeuchi^1^, Nozomi Matsumura^1,2,^ Tomoko Ohno^1^, Takeshi Fujita^2^, Mizuki Asano^2^, Nobuhisa Mizuki^1^

1. Department of Ophthalmology and Visual Science, Yokohama City University Graduate School of Medicine
2. Department of Ophthalmology, Kanagawa Children’s Medical Center

**Supplementary Fig. S1.** Classification of ciliary touch (right eye).

**Supplementary Fig. S2.** Comparison of changes in astigmatism for both pre- and post-surgery for epiblepharon.

**Supplementary Fig. S3.** Pre- and post-operative photographs of representative cases

**Supplementary Fig. S1.** Classification of ciliary touch (right eye). Modified from Lee et al.^11^

From the left, grade 1: mild, grade 2: moderate, and grade 3: severe. Ciliary touch was assessed in the primary eye position.


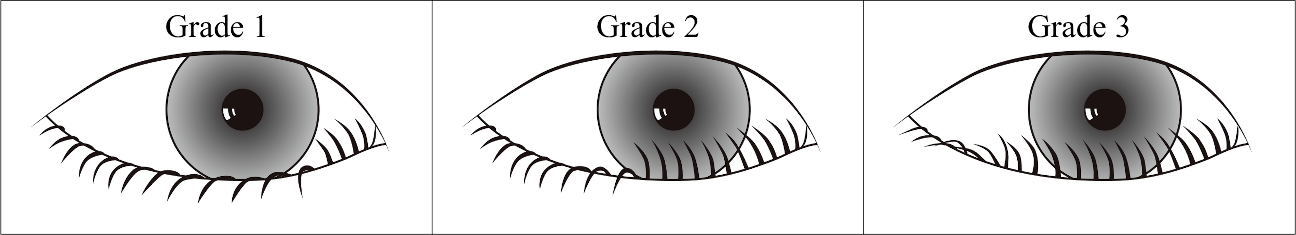


**Supplementary Fig. S2.** Comparison of changes in astigmatism for both pre- and post-surgery for epiblepharon.

The improvement in astigmatism at 6 months after surgery was significantly higher in the incisional surgery group compared with the nonincisional surgery group (P = 0.008).

D, diopter


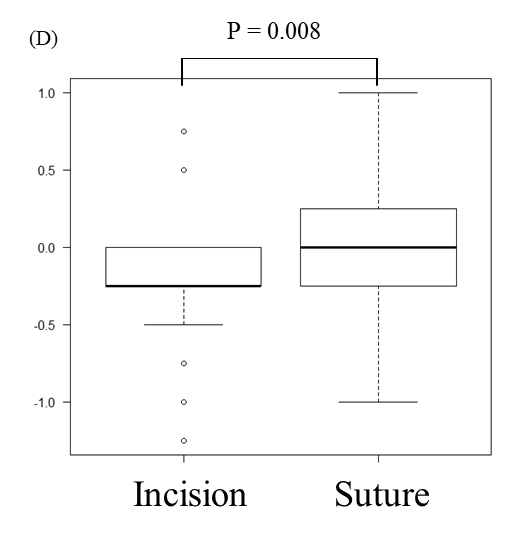


**Supplementary Fig. S3.** Pre- and post-operative photographs of representative cases.

A. Before (upper) and 6 months after performing (lower) incision techniques (modified hotz method with lid margin splitting)

B. Before (upper) and 6 months after performing (lower) suture techniques (buried suture)

A


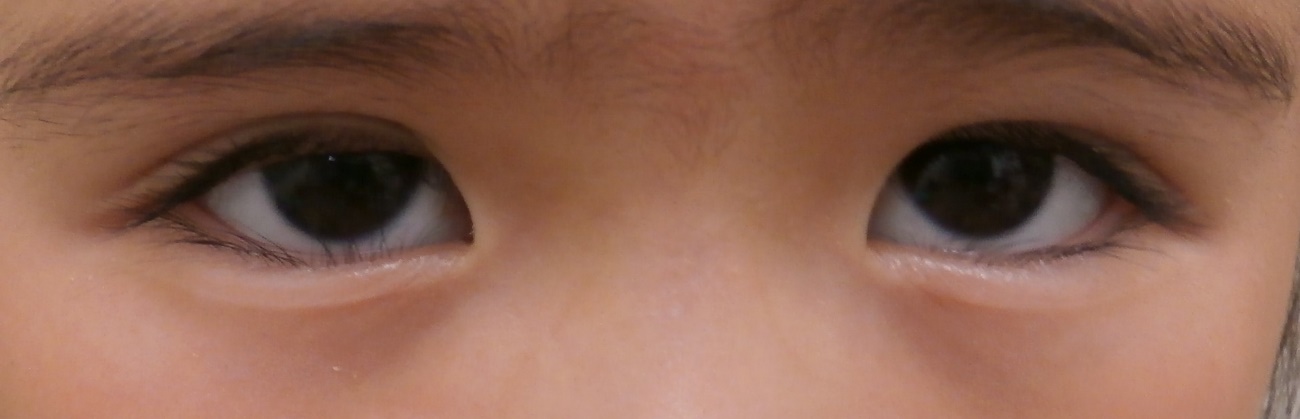


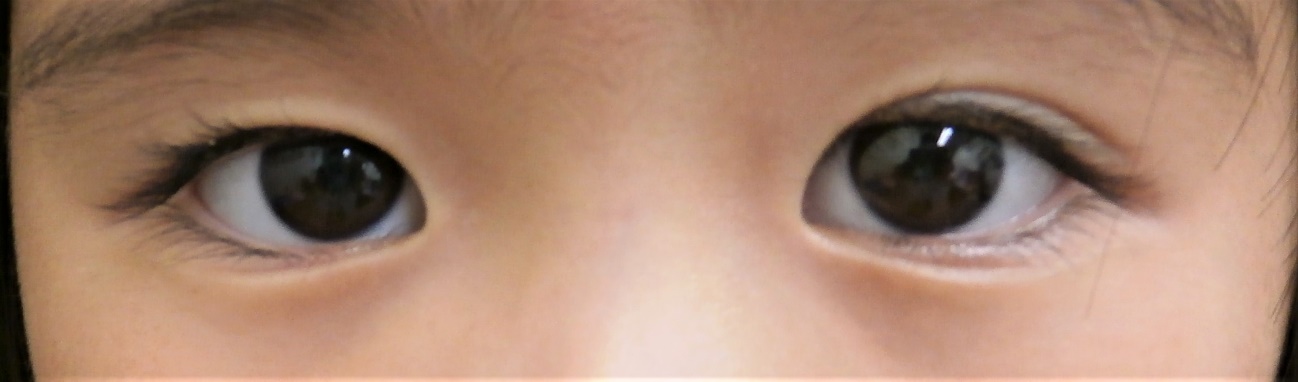


B
